# Prime editing in mice reveals the essentiality of a single base in driving tissue-specific gene expression

**DOI:** 10.1186/s13059-021-02304-3

**Published:** 2021-03-16

**Authors:** Pan Gao, Qing Lyu, Amr R. Ghanam, Cicera R. Lazzarotto, Gregory A. Newby, Wei Zhang, Mihyun Choi, Orazio J. Slivano, Kevin Holden, John A. Walker, Anastasia P. Kadina, Rob J. Munroe, Christian M. Abratte, John C. Schimenti, David R. Liu, Shengdar Q. Tsai, Xiaochun Long, Joseph M. Miano

**Affiliations:** 1 Department of Medicine, Vascular Biology Center, Medical College of Georgia at Augusta University, Augusta, GA 30912 USA; 2 Department of Hematology, St. Jude Children’s Research Hospital, Memphis, TN 38195 USA; 3 Merkin Institute of Transformative Technologies in Healthcare, Broad Institute of MIT and Harvard, Cambridge, MA 02142 USA; 4 Department of Chemistry and Chemical Biology, Harvard University, Cambridge, MA 02138 USA; 5 Howard Hughes Medical Institute, Harvard University, Cambridge, MA 02138 USA; 6 Department of Physiology, Albany Medical College, Albany, NY 12208 USA; 7 Synthego Corporation, Redwood City, CA 94025 USA; 8 Department of Biomedical Sciences, Cornell University, Ithaca, NY 14853 USA

**Keywords:** Mouse, CRISPR, Prime editing, Genome editing, Transcription, Gene expression

## Abstract

**Background:**

Most single nucleotide variants (SNVs) occur in noncoding sequence where millions of transcription factor binding sites (TFBS) reside. Here, a comparative analysis of CRISPR-mediated homology-directed repair (HDR) versus the recently reported prime editing 2 (PE2) system was carried out in mice over a TFBS called a CArG box in the *Tspan2* promoter.

**Results:**

Quantitative RT-PCR showed loss of *Tspan2* mRNA in aorta and bladder, but not heart or brain, of mice homozygous for an HDR-mediated three base pair substitution in the *Tspan2* CArG box. Using the same protospacer, mice homozygous for a PE2-mediated single-base substitution in the *Tspan2* CArG box displayed similar cell-specific loss of *Tspan2* mRNA; expression of an overlapping long noncoding RNA was also nearly abolished in aorta and bladder. Immuno-RNA fluorescence in situ hybridization validated loss of *Tspan2* in vascular smooth muscle cells of HDR and PE2 CArG box mutant mice. Targeted sequencing demonstrated variable frequencies of on-target editing in all PE2 and HDR founders. However, whereas no on-target indels were detected in any of the PE2 founders, all HDR founders showed varying levels of on-target indels. Off-target analysis by targeted sequencing revealed mutations in many HDR founders, but none in PE2 founders.

**Conclusions:**

PE2 directs high-fidelity editing of a single base in a TFBS leading to cell-specific loss in expression of an mRNA/long noncoding RNA gene pair. The PE2 platform expands the genome editing toolbox for modeling and correcting relevant noncoding SNVs in the mouse.

**Supplementary Information:**

The online version contains supplementary material available at 10.1186/s13059-021-02304-3.

## Background

Proper spatiotemporal control of gene expression requires the RNA polymerase II complex to physically associate with DNA-binding transcription factors and their coregulators over transcription factor binding sites (TFBS) located in the promoter and enhancer region of target genes [1]. Elucidating enhancer function and the role of individual TFBS has informed our understanding of basic mechanisms underlying gene transcription as well as the development of *Cre*/*lox*P mouse models for cell-restricted gene inactivation. Since most sequence variants (e.g., single nucleotide variants or SNVs) associated with human disease occur in noncoding sequence space where TFBS reside [2, 3], understanding the biology of TFBS in orchestrating gene transcription may provide insight into basic mechanisms of disease [4]. Traditionally, TFBS function was studied in in vitro or in vivo reporter assays, outside of their normal genomic context. Notably, few TFBS have been modified in their native genomic milieu of the mouse and nearly all yielded imprecise mutations and genomic scarring (e.g., residual *lox*P sequence) [5–8]. Generating such mouse models with conventional embryonic stem cell targeting is labor-intensive and expensive, and the results can be uncertain given the known redundancies in TFBS utilization for target gene transcription [9].

The repurposing of the bacterial clustered regularly interspaced short palindromic repeats (CRISPR) system as a programmable, RNA-directed DNA endonuclease [10, 11] has greatly simplified and accelerated precision editing of the mouse genome [12–14]. The first generation of CRISPR editing in mice utilized three components: an endonuclease (Cas9); a single-guide RNA (sgRNA) that shepherds Cas9 to the sequence to be edited; and a repair template, generally a single-strand oligodeoxynucleotide (ssODN) engineered to carry small insertions, deletions, or substitutions that are incorporated into the target DNA sequence during homology-directed repair (HDR) of the sgRNA-Cas9 induced double-strand break [15–17]. Three-component CRISPR successfully disrupted TFBS in their native genomic context of mice, revealing insight into target gene expression in an in vivo setting [18–20]. However, HDR-mediated editing is often inefficient, is limited to actively dividing cells, is associated with unwanted collateral indel mutations, and may yield off-targeting events [21]. A second-generation CRISPR platform, called base editing [22], was developed wherein an sgRNA directs a Cas9 nickase fused to a cytidine or adenine deaminase to target DNA for installation of base substitutions without the generation of a double-strand break in DNA or the need of a repair template, thus simplifying delivery and mitigating the proportion of indels. This two-component platform was used to edit separable TFBS in the mouse with no detectable off-targets [23]. However, base editing is currently limited to base transitions and may generate so-called bystander substitutions at neighboring bases within the editing window, thereby complicating the identification of correctly edited TFBS. Recently, a new two-component genome editing platform, called prime editing, was developed wherein a Cas9 nickase fused to an engineered Maloney murine leukemia virus reverse transcriptase can directly copy desired edits to the target DNA sequence from a prime editing guide RNA (pegRNA) [24]. Prime editing installed > 175 different edits, including all possible base substitutions, in various human cell lines with limited off-target events [24]. Thus, in principle, prime editing represents a versatile, precision-guided platform that can potentially correct all SNVs of clinical importance [24]. Prime editing has been reported in plants [25–27], in early-staged mouse embryos [28, 29], and in Drosophila [30]. However, there has yet to be a comparative analysis of prime editing versus CRISPR-mediated HDR editing in mice bred through the germline and analyzed for phenotypes. Here, we sought to test the efficiency of prime editing versus three-component HDR editing at a single TFBS in mice. Results demonstrate high-fidelity in vivo prime editing and an unexpected phenotype in mice carrying a single-base substitution within a TFBS.

## Results

### Three-component HDR editing of a CArG box in the mouse *Tspan2* promoter

**Fig. 1:**
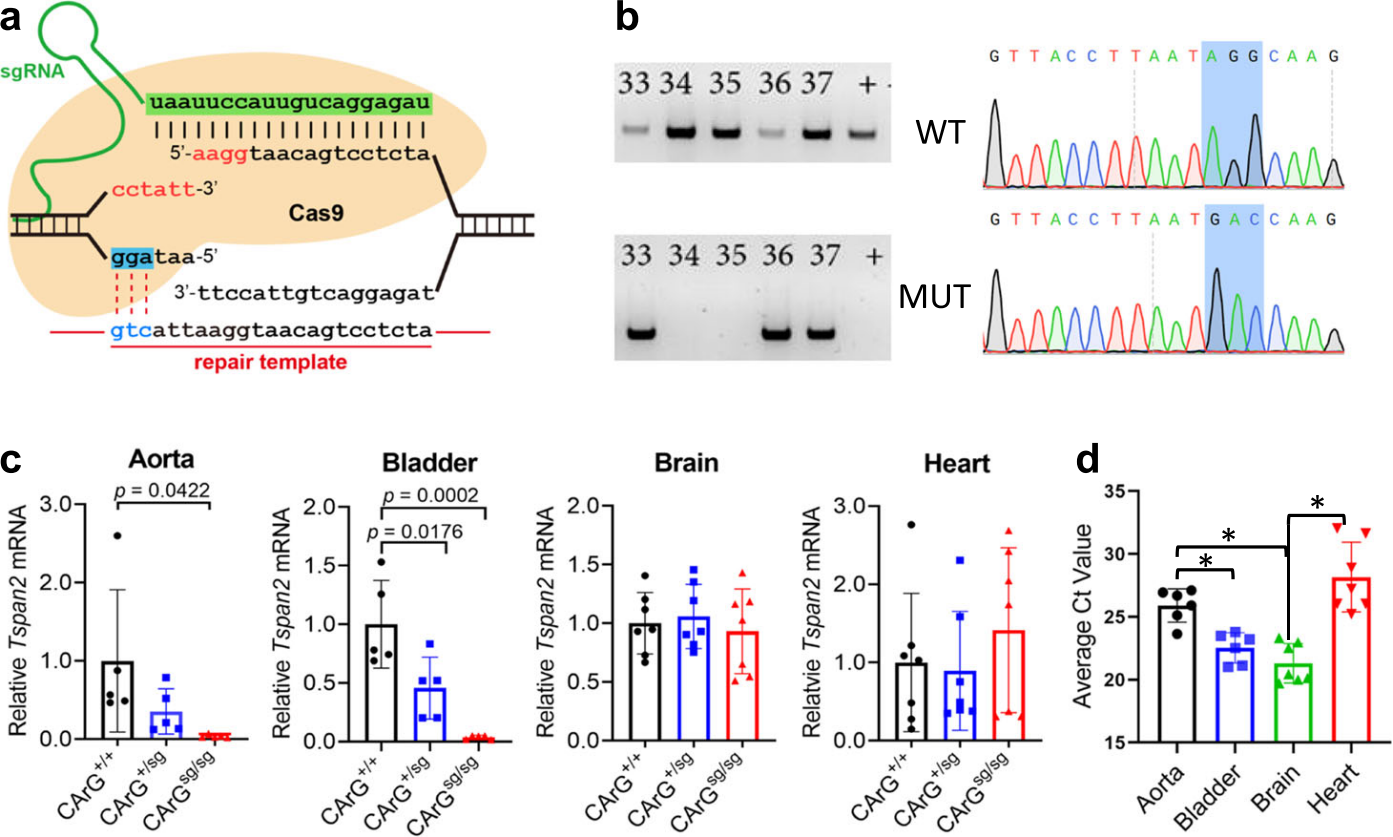
HDR-mediated editing of *Tspan2* CArG box. **a** Targeting strategy with CArG box sequence (red), protospacer sequence (green), PAM sequence (blue shade), and 3 bp substitution (blue) in ssODN repair template. **b** Allele-specific PCR of some founder mice with evidence of wild type (top) or mutant (bottom) edit and corresponding Sanger sequencing of CArG box (shaded). **c** qRT-PCR of *Tspan2* in indicated tissues and genotypes (*n* = 5–7 mice/genotype). Black, blue, and red bars here and below represent relative (wild type set to value of 1) mean *Tspan2* mRNA (± STD) in wild type, heterozygous, and homozygous genotypes, respectively. **d** Average Ct values for indicated tissues (*n* = 6–7 mice/tissue). Asterisks indicate *p* < 0.05. sg, sgRNA edited *Tspan2* CArG box

**Fig. 2:**
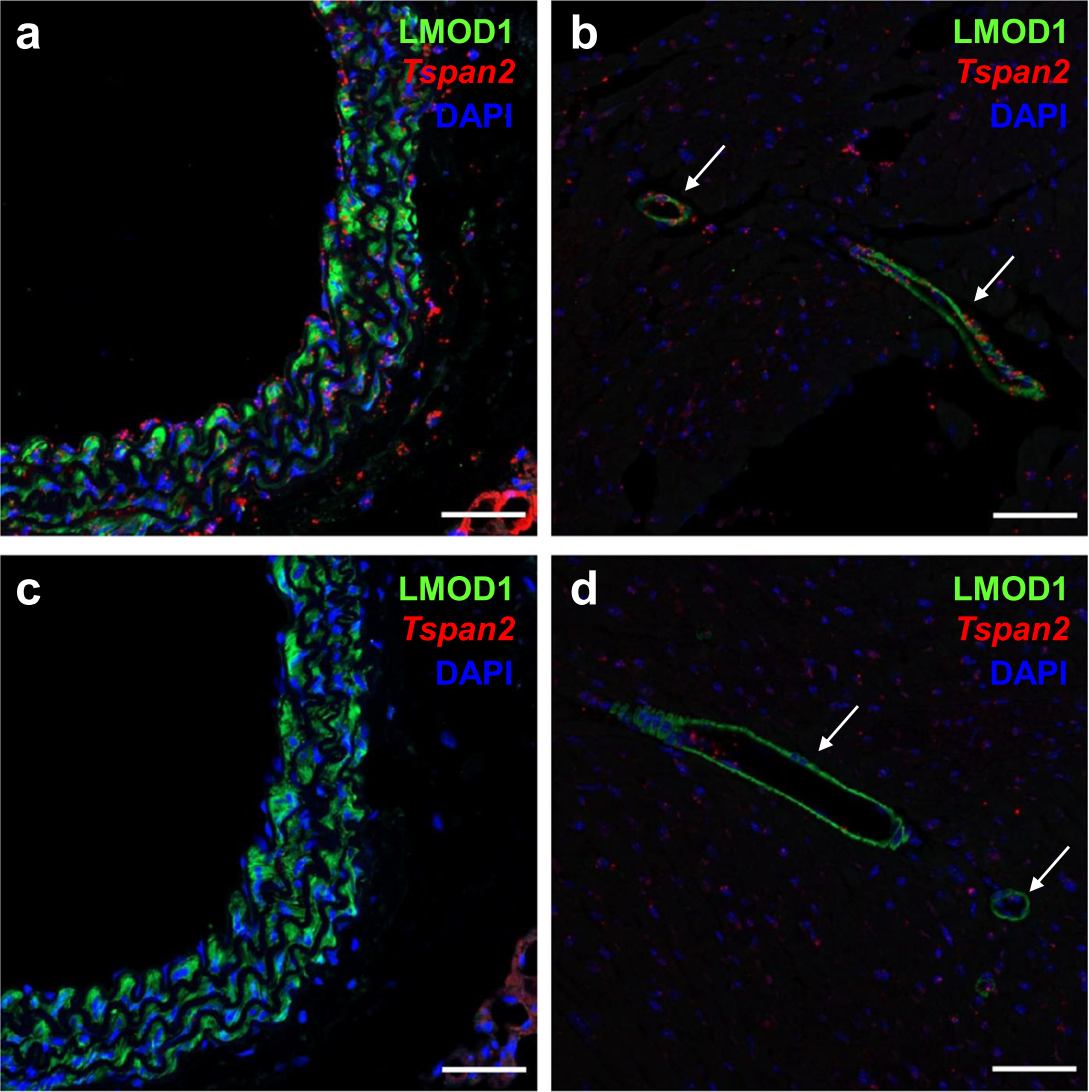
Spatial localization of *Tspan2* mRNA in HDR-edited mouse tissues. Immunofluorescence (LMOD1) and RNA FISH (*Tspan2*) of wild type aorta (**a**) and heart (**b**) versus *Tspan2*^*sg/sg*^ CArG box mutant (HDR) aorta (**c**) and heart (**d**). Arrows point to coronary vessels of the heart. Note decrease in *Tspan2* mRNA (red dots) in vascular smooth muscle cells (labeled green with LMOD1 antibody) of aorta and coronary vessels in CArG box mutants (**c, d**). Scale bars are 50 μm. Representative images from *n* = 3 mice

The CArG box is a TFBS for serum response factor (SRF), a widely expressed TF that directs disparate programs of gene expression [31]. An SRF-binding CArG box is located 539 base pairs upstream of the major transcription start site of the human *TSPAN2* locus (Additional file 1: Supplementary Fig. 1a). This CArG box is conserved in many mammalian species, including mouse (Additional file 1: Supplementary Fig. 1b). Previous results demonstrated in vitro activity of this CArG box [32]; however, whether this TFBS is necessary for *Tspan2* expression in mice is unknown. To address this question, a sgRNA overlapping the CArG box was designed with the CRISPOR tool [33]. The CArG box sequence (CCW_6_GG) begins and ends with a protospacer adjacent motif (NGG), making this class of TFBS an ideal sequence for HDR-mediated genome editing (Additional file 1: Supplementary Fig. 1b). An ssODN with three nucleotide substitutions expected to disrupt SRF binding to the CArG box was also designed (Fig. 1a). The Cas9 protein, sgRNA, and ssODN were injected into 204 mouse zygotes. Following overnight culture, 143/204 (70%) viable two-cell staged mouse embryos were transferred to 5 recipient females and 37/143 (26%) live-born births were obtained. Allele-specific PCR genotyping revealed 20/37 (54%) founder mice with evidence of correct editing, with 4/20 (20%) showing obvious indels (Additional file 1: Supplementary Fig. 2 and Fig. 1b). Due to the large number of PCR-positive mice, 11/20 founders were selected for on-target and off-target analyses (Additional file 1, Supplementary Fig. 2). The three base pair substitution was validated in a founder mouse by Sanger sequencing (Fig. 1b) and bred for germline transmission of the mutant CArG box. Next, heterozygous F_1_ mice were inter-crossed to generate homozygous *Tspan2* CArG mutant mice (*Tspan2*^*sg/sg*^). Normal Mendelian ratios were observed (12 *Tspan2*^*+/+*^, 27 *Tspan2*^sg/+^, and 15 *Tspan2*^sg/sg^). Several tissues were isolated from each genotype for qRT-PCR analysis. Compared to *Tspan2*^*+/+*^ controls, expression of *Tspan2* mRNA in *Tspan2*^sg/sg^ mice was sharply attenuated in smooth muscle-rich tissues of aorta and bladder (Fig. 1c). An intermediate level of *Tspan2* mRNA was seen in *Tspan2*^sg/+^ mice suggesting bi-allelic expression (Fig. 1c). Although *Tspan2* mRNA in heart and brain is, respectively, similar to or more abundant than *Tspan2* in aorta (Fig. 1d), little change in expression was detected in heart or brain of heterozygous or homozygous CArG mutant mice (Fig. 1c). Repeated attempts to show TSPAN2 protein by Western blotting or immunofluorescence microscopy were unsuccessful. Accordingly, immunofluorescence microscopy of a smooth muscle cell marker (LMOD1) [34] was combined with RNA fluorescence in situ hybridization to confirm loss of *Tspan2* mRNA in smooth muscle cells of the aorta and coronary arteries of the heart (Fig. 2). Collectively, these findings demonstrate the critical role of a single TFBS for cell-specific expression of *Tspan2* in adult mice.

### Two-component prime editing of a CArG box in the mouse *Tspan2* promoter

**Fig. 3:**
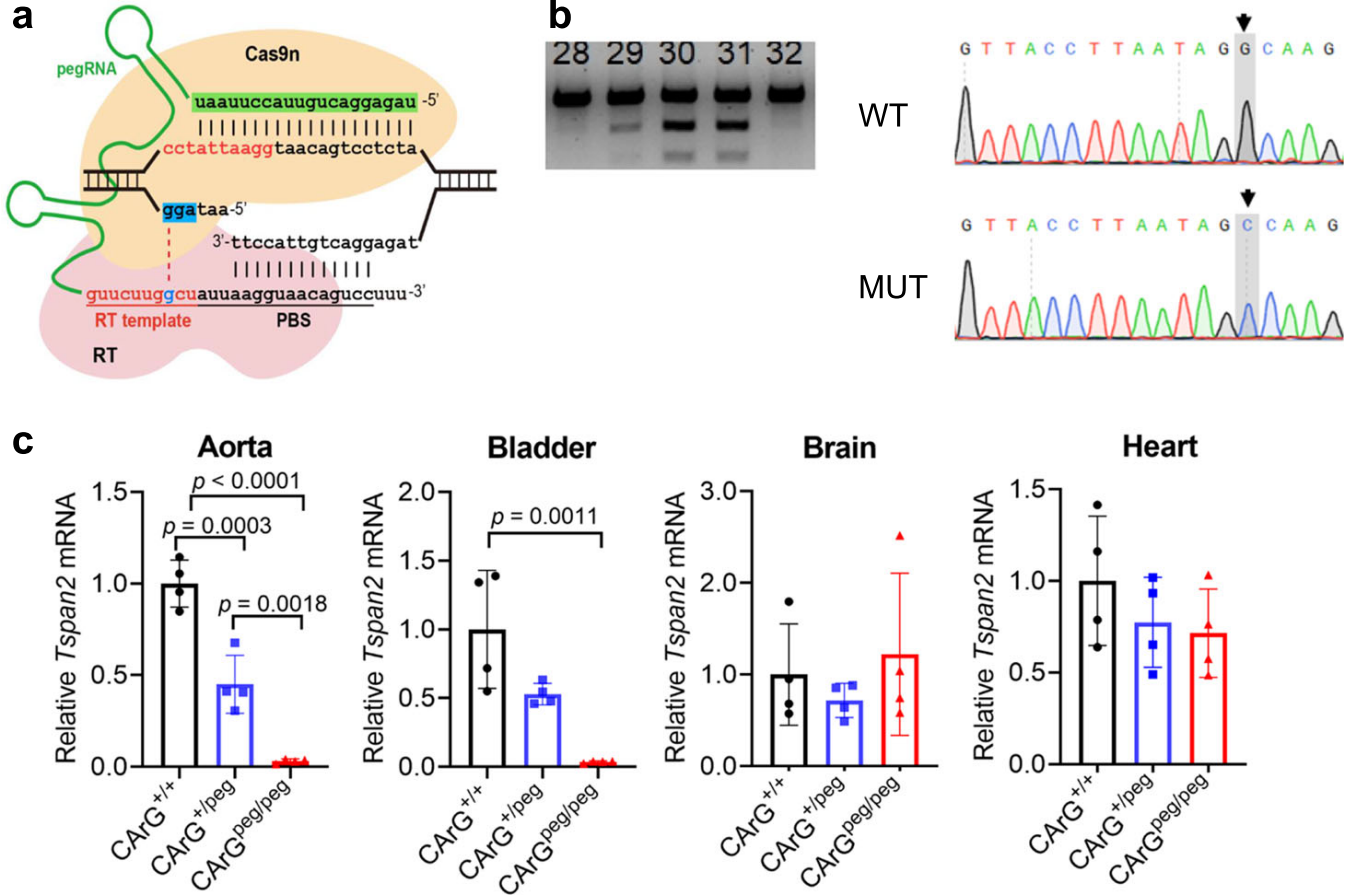
PE2-mediated editing of *Tspan2* CArG box. **a** Targeting strategy with CArG box in red sequence, protospacer in green sequence, PAM in blue shaded sequence, and 1 bp substitution within RT template in blue. **b** Representative genotyping of several founders with correct installation of 1 bp transversion (left) and Sanger sequencing of CArG box showing correct edit in a mutant founder (right, shaded). **c** qRT-PCR of *Tspan2* mRNA in indicated tissues and genotypes (*n* = 4 mice per genotype). peg, pegRNA edited mouse; RT, reverse transcriptase; PBS, primer binding site

**Fig. 4:**
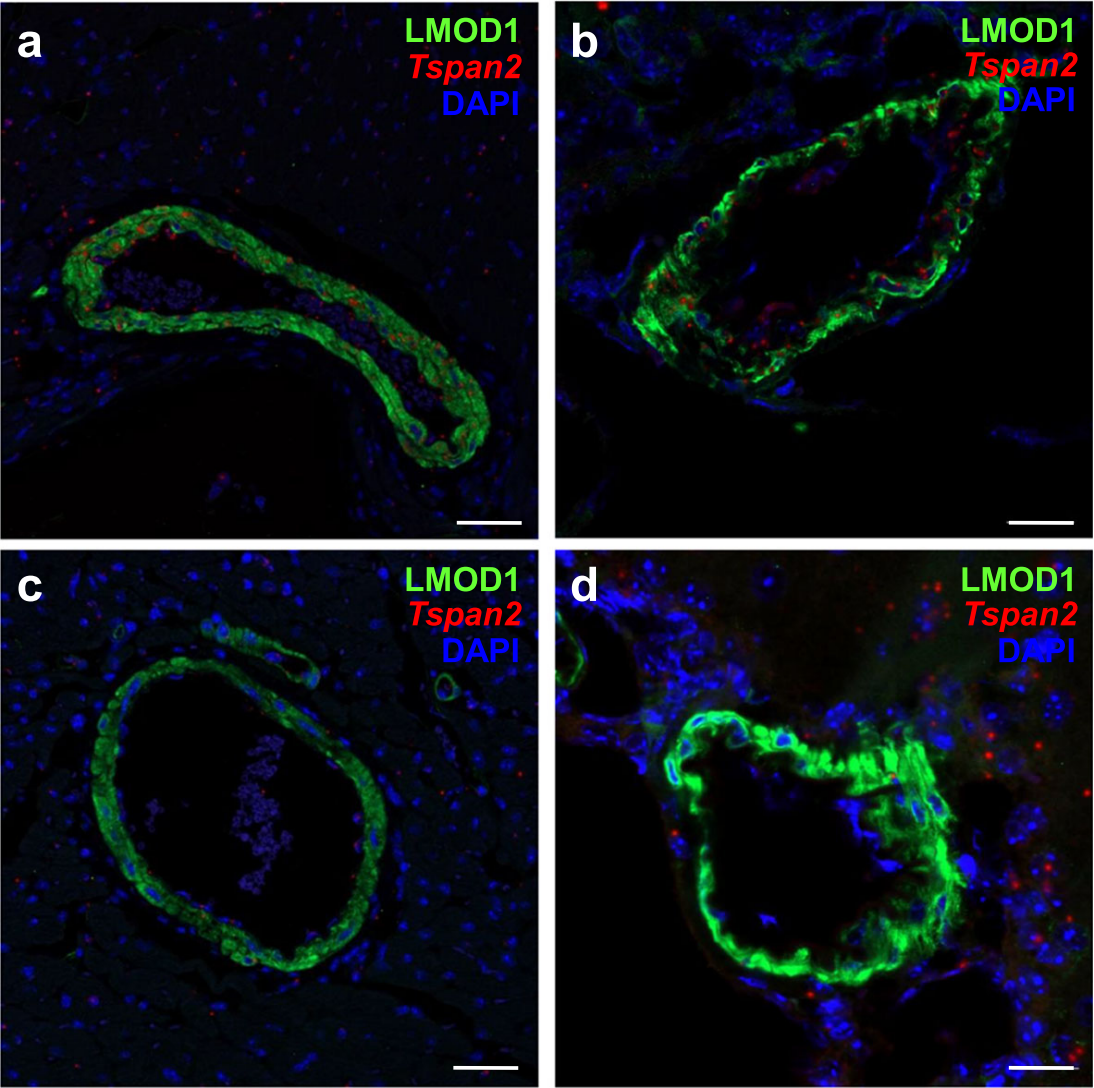
Spatial localization of *Tspan2* mRNA in PE2-edited mouse tissues. Immunofluorescence (LMOD1) and RNA FISH (*Tspan2*) of blood vessels in wild type heart (**a**) and brain (**b**) versus *Tspan2*^*peg/peg*^ CArG box mutant heart (**c**) and brain (**d**). The *Tspan2* mRNA is indicated by the red dots in vascular smooth muscle cells (labeled green with LMOD1 antibody) of wild type blood vessels (**a, b**), but is nearly absent in CArG mutant vessels (**c, d**). Scale bars are 50 μm. Representative images from *n* = 2 mice

Inspired by previous in vitro studies demonstrating an attenuation in SRF binding to CArG boxes carrying single-base pair substitutions [35], we set out to use the recently described prime editing platform [24] to target the same CArG box of the *Tspan2* promoter. Several prime editing (PE) plasmids carrying the Cas9 nickase fused to reverse transcriptase exist, but the PE2 plasmid was selected since this version of prime editor showed low-level indels in cultured cells [24]. Optimal in vitro transcription of pCMV-PE2 [24] was achieved by extending the incubation time to 3 h and treating samples with RNase inhibitors (Additional file 1, Supplementary Fig. 3). A synthetic pegRNA was generated with the following sequence features: the same 20 nucleotide protospacer sequence used for the HDR experiment above followed by the scaffold extended with 10 nucleotides corresponding to the reverse transcriptase template and a 16-nucleotide primer binding site (Fig. 3a). A single-base substitution, a C>G transversion, was engineered at position + 8 of the reverse transcriptase template (Fig. 3a). In vitro-transcribed PE2 mRNA and synthetic pegRNA were injected into 234 mouse zygotes. Following overnight culture, 175/223 (78%) viable two-cell staged embryos were transferred to 5 recipient females and 47/175 (27%) live-born births were obtained. Restriction digestion of a PCR product from each live-born pup revealed 12/47 (26%) founder mice with evidence of correct editing (Additional file 1: Supplementary Fig. 4 and Fig. 3b). Sanger sequencing of a founder mouse showed precise incorporation of the C>G transversion (Fig. 3b). Two of the sequence-confirmed PE2 founders transmitted the edited allele through the germline for heterozygous intercrossing. Normal Mendelian ratios were seen in F_1_ pegRNA mice (12 *Tspan2*^*+/+*^, 24 *Tspan2*^peg/+^, and 10 *Tspan2*^peg/peg^). Remarkably, the expression of *Tspan2* mRNA was virtually abolished in aorta and bladder of *Tspan2*^peg/peg^ adult mice with little change in brain and heart (Fig. 3c). Similar to the *Tspan2*^sg/sg^ mice above, RNA fluorescence in situ hybridization of *Tspan2*^peg/peg^ mice demonstrated loss of *Tspan2* mRNA in smooth muscle cells of blood vessels in brain and heart (Fig. 4). *Tspan2*^sg/sg^ and *Tspan2*^peg/peg^ mice were normal and fertile and showed no evidence of pathology.

**Fig. 5:**
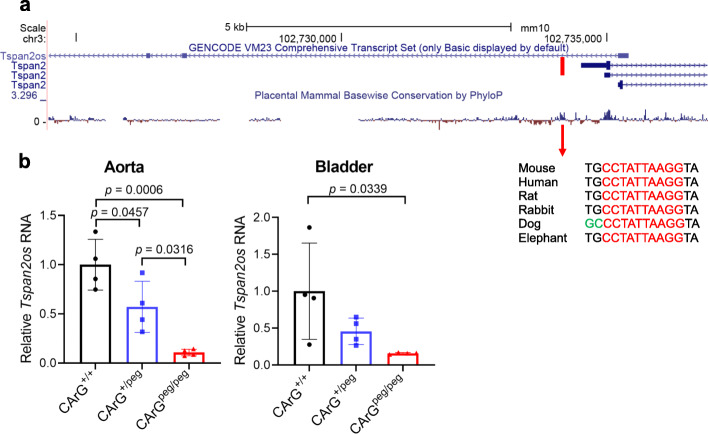
Mouse *Tspan2* and *Tspan2os* loci. **a** UCSC Genome Browser screenshot of the 5′ mouse *Tspan2* locus and overlapping, divergently transcribed lncRNA, *Tspan2os*. The sequence of the CArG box shown here is the complement of that shown in Supplementary Fig. 1 due to direction of transcription in mouse versus human *Tspan2*. Note the CArG box falls within a high degree of mammalian conservation (red arrow). **b** qRT-PCR of *Tspan2os* RNA in aorta and bladder of indicated PE2-mediated genotypes. *n* = 4 aortae for each genotype

An antisense long noncoding RNA (LncRNA), called *Tspan2os*, overlaps the *Tspan2* locus in the mouse genome (Fig. 5a). The transcription start site of *Tspan2os* is located 648 base pairs downstream of the *Tspan2* start site, and the putative promoter of *Tspan2os* is ~ 900 base pairs 5′ of the CArG box (Fig. 5a). Given such close proximity, we surmised that expression of *Tspan2os* would be similarly dependent on the targeted CArG box. Indeed, qRT-PCR revealed near abrogation of *Tspan2os* RNA in aorta and bladder of mice homozygous for the C>G transversion (Fig. 5b). Taken together, these findings demonstrate an essential role of a single base within a TFBS in co-regulating an mRNA-LncRNA antisense gene pair.

### On-target editing fidelity in HDR versus prime edited founder mice

**Fig. 6:**
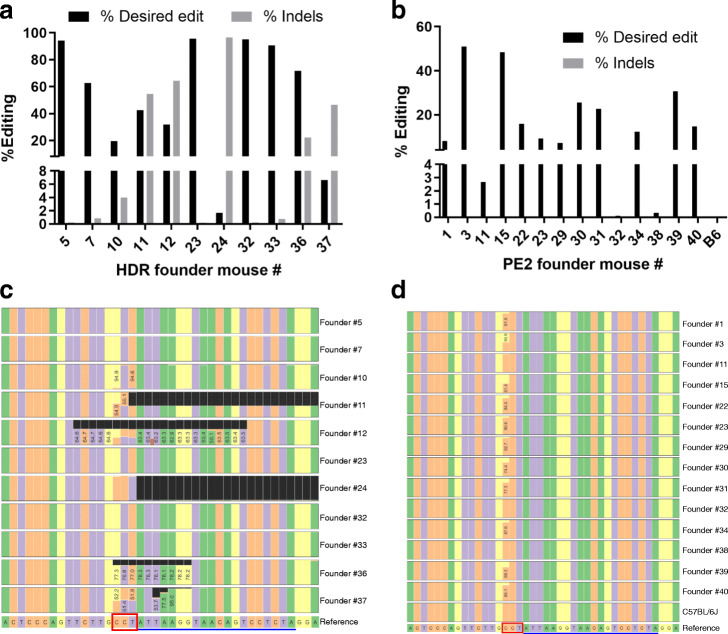
On-target sequence fidelity at the *Tspan2* CArG box. Percent editing across HDR (**a**) and PE2 (**b**) founder mice. CRISPResso sequence output for individual founders from sgRNA (**c**) and pegRNA (**d**) study. Protospacer (blue line) and PAM (red box) are indicated as are numbers indicating frequency of correct edits. Black boxes in panel **c** indicate deletions. Please note, a 150 bp deletion could not be aligned in CRISPResso for HDR founders 7, 10, and 33, but is included in the quantitative data of panel **a**. PE2 founders 32 and 38 represent littermate controls that did not exhibit on-target editing

Genome editing with wild type Cas9 can elicit undesired editing outcomes such as indels among a large fraction of edited cells [36]. On the other hand, prime editing, particularly with the single-nick PE2 system, yields a much higher purity of edited products [24]. Accordingly, targeted sequencing analysis on genomic DNA derived from the spleen of 11 HDR and 12 PE2 founder mice was performed to evaluate the fidelity of on-target editing. The mean percentage of sequencing reads with correct on-target editing was 55.65% (range 1.67–95.56%) for HDR founder mice (Fig. 6a) versus 20.74% (range 2.66–50.94%) for PE2 founder mice (Fig. 6b; Additional file 1: Supplementary Fig. 5). Despite a significantly higher frequency of on-target editing, all of the HDR founders showed undesired indels (mean of 40.11%, range 0.91–93.91%) (Fig. 6a). In contrast, none of the PE2 founders displayed indels above background at the on-target editing site (Fig. 6b). CRISPResso analysis further documented the frequency of indels in each of the founder mice (Fig. 6c, d). These results demonstrate precise PE2-mediated on-target editing, with no spurious indels, in the C>G transversion of the *Tspan2*/*Tspan2os* CArG box.

### Off-target editing in HDR versus prime edited founder mice

**Fig. 7:**
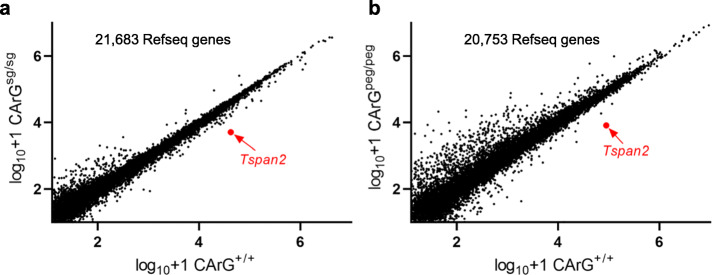
Bulk RNA-seq of aortae from HDR and PE2-edited mice. Scatter plots between **a** HDR (sgRNA) and **b** PE2 (pegRNA) mice. The position of differential *Tspan2* normalized reads is indicated in red. There was no overlap in genes up- or downregulated between the HDR and PE2 scatter plots. Many of the upregulated transcripts, particularly in the pegRNA experiment, are due to large deviations in reads among single replicates. For a listing of the significantly regulated genes, please see Supplementary Table S2. *n* = 4 aortae for each genotype

There are > 1200 permutations of the CArG box across mammalian genomes [37]. Because the *Tspan2*/*Tspan2os* CArG box encompasses the PAM and PAM proximal protospacer sequence, we considered the possibility of inadvertent targeting of other CArG boxes by either HDR- or PE2-mediated editing and, if present near a target gene, reduced gene expression as shown here for the *Tspan2/Tspan2os* gene pair. CRISPOR analysis of the protospacer sequence targeting the CArG box revealed specificity scores of 80 (MIT) and 93 (CFD) and 0, 1, 0, 12, and 68 predicted off-targets with 0, 1, 2, 3, or 4 mismatches, respectively (Additional file 1, Supplementary Fig. 6). Of the 53/81 (65%) CRISPOR predicted off-targets harboring a potential SRF-binding CArG box, only 7/53 (13%) are located within four kilobases of the transcription start site where all known functional CArG boxes reside (Additional file 2: Table S1) [37]. To address whether the CArG box edits had distal effects on gene expression or off-targeting events that did not segregate upon breeding leading to local gene repression similar to *Tspan2/Tspan2os*, bulk RNA-seq analysis was carried out on aortae from *Tspan2*^*+/+*^ versus either *Tspan2*^*sg/sg*^ or *Tspan2*^*peg/peg*^ mice [38]. No distal effect on gene expression was evident (Fig. 7) nor were decreases in expression of genes adjacent to the 81 CRISPOR predicted off-targets (Additional file 2: Table S1). Moreover, the only target genes significantly reduced in HDR (*Tspan2*^*sg*/sg^) and PE2 (*Tspan2*^*peg/peg*^) mice were *Tspan2* and *Tspan2os*, both of which showed ~ 90% decrease versus *Tspan2*^*+/+*^ aorta (Fig. 3c and Fig. 5b). Several significantly changed genes in *Tspan2*^*sg*/sg^ or *Tspan2*^*peg/peg*^ mice harbor proximal CArG boxes that were not identified by CRISPOR; however, only one of these (*Hist2h2be*) was downregulated in mutant aorta (Additional file 3: Table S2). Of note, the two conserved CArG boxes and flanking sequence of *Hist2h2be* have 13 and 17 mismatches with the protospacer, making them unlikely targets for the sgRNA or pegRNA.

**Fig. 8:**
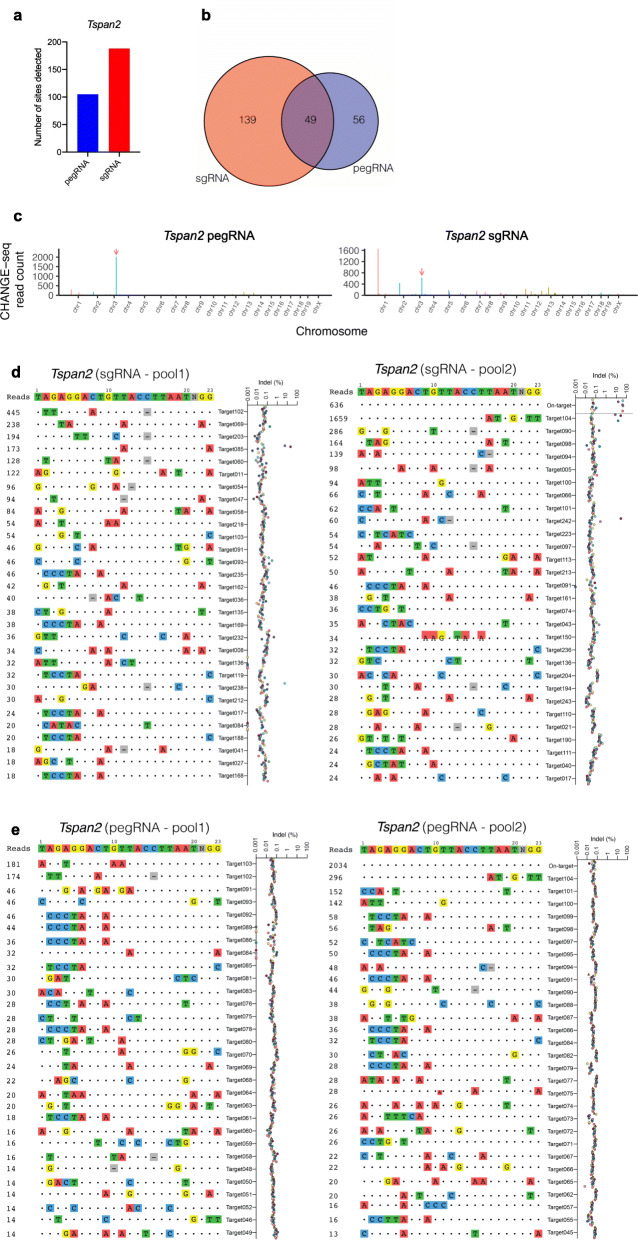
Genome-wide off-target analysis of HDR and PE2 edited founder mice. **a** Bar plot of number of CHANGE-seq sites detected using Cas9 WT and synthetic sgRNA or pegRNA targeting *Tspan*2, on WT genomic DNA from same strain of mice used in HDR and PE2 editing experiments. **b** Venn diagram depicting common predicted off-target sites for sgRNA (orange) and pegRNA (blue) groups. **c** Manhattan plots of CHANGE-seq detected on- and off-target sites organized by chromosomal position, for sgRNA and pegRNA, with bar heights representing CHANGE-seq read count. Arrow indicates the on-target site. **d, e** Indel frequencies evaluated by rhAmpSeq at on- and off-target sites detected by CHANGE-seq for sgRNA founders (**d**) and for pegRNA founders (**e**)

To assess off-targeting events in HDR versus PE2 edited mice with a sensitive, unbiased genome-wide method, the recently described *c*ircularization for *h*igh-throughput *a*nalysis of *n*uclease *g*enome-wide *e*ffects by *seq*uencing (CHANGE-seq) was utilized with wild type Cas9 nuclease [39]. CHANGE-seq revealed 105 and 188 predicted off-targets for Cas9 complexed with pegRNA or sgRNA, respectively (Fig. 8a). 21/81 (26%) CRISPOR predicted off-targets overlapped with those derived from CHANGE-seq, and of the CHANGE-seq off-targets overlapping in both sgRNA and pegRNA samples (Fig. 8b), only 2/49 (4%) were found in the CRISPOR pool. Next, each of the HDR and pegRNA founder mice was interrogated for evidence of unintended off-target mutations at a total of 244 target sites predicted with CHANGE-seq and in 13 CasOFFinder [40] sites using rhAmpSeq, an approach previously validated for concordance with standard targeted sequencing [39]. Off-target mutations were observed at relatively high frequencies of approximately 5.5 to 60.2% across five sites in 5/11 HDR founder mice (Fig. 8c, right and Fig. 8d and Additional files 4 and 5: Tables S3 and S4). In contrast, no detectable off-target mutations with frequencies above background were seen in pegRNA founders (Fig. 8c, left and Fig. 8e and Additional files 4 and 5: Tables S3 and S4). Further, no mutations above background were observed in control mice from the same colony.

We next analyzed whether any of the 244 CHANGE-seq sites contain a CArG box. There are 66/105 (63%) and 109/188 (58%) CArG boxes in the pegRNA and sgRNA CHANGE-seq candidate off-target sites, respectively. The vast majority of these sites (171/175 or 98%) are distal (> 4 kb) from any annotated transcript. Nevertheless, we assessed whether the nearest transcription unit in these distal sites exhibited changes in bulk RNA-seq of aorta; none showed a significant decrease in expression (Additional files 6 and 7: Tables S5 and S6). Only 4/175 (2%) CHANGE-seq targets have a CArG box in close proximity to the transcription start site, but none showed any change in bulk RNA-seq of aorta (Additional files 6 and 7: Tables S5 and S6). Taken together, these analyses demonstrate that while there were more off-targeting events in the HDR founders (5/11 or 45%), none resulted in a CArG box-dependent decrease in gene expression. It should be noted, however, that the bulk RNA-seq studies were necessarily performed in germline-transmitted mice where potentially deleterious off-targets that could affect CArG-dependent gene expression may have segregated from the *Tspan2* CArG mutation.

## Discussion

The prime editing 2 (PE2) platform initially demonstrated versatility of editing in cultured cells with minimal off-targeting events [24]. It is essential, however, to extend findings to more complex model systems and compare the relative efficiency of PE2 with other genome editing platforms such as base editing and HDR-mediated editing. Recent work in mouse embryos has shown variable fidelity of prime editing, depending on the prime editing system used [28, 29]. However, no studies have yet to compare prime editing with conventional HDR in an animal model using the same protospacer sequence, and there have been no reported animal phenotypes following prime editing. Here, the PE2 system [24] was tested and compared to conventional HDR-mediated editing in mice using the same protospacer targeting a TFBS (CArG box) located in the *Tspan2* promoter. *Tspan2* encodes for a membrane-associated protein highly enriched in vascular and visceral smooth muscle cell-containing tissues [32]. While both editing platforms successfully installed the engineered nucleotide substitutions within the CArG box, PE2 did so without measurable on-target indels or off-targeting events. HDR-mediated installation of a three-base substitution in the CArG box resulted in high efficiency incorporation of the desired edit and near loss of *Tspan2* mRNA expression in smooth muscle cells; however, all founder mice exhibited on-target indels and several exhibited off-target editing events. Prime editing of a single nucleotide within the CArG box resulted in ~ 90% reduction in expression of *Tspan2* in vascular and visceral smooth muscle tissues of adult mice; little change in expression was observed in heart muscle or in brain. A similar reduction in RNA expression was observed for an overlapping LncRNA (*Tspan2os*). To our knowledge, this is the first demonstration of the essentiality of a single base within a TFBS for tissue-restricted gene expression in mice and the co-regulation of an mRNA/LncRNA gene pair.

The severe attenuation of *Tspan2* in aorta and bladder provides a unique opportunity to elucidate TSPAN2 protein function in smooth muscle without the need for engineering complex Cre/*lox*P mice that restrict loss-of-function of a gene to smooth muscle-containing tissues. Moreover, since the coding sequence of *Tspan2* was unadulterated, future genetic rescue studies could be simplified using CRISPR activation [41] to override the regulatory edit in the CArG box. One caveat of this approach, however, relates to mRNA/LncRNA gene pairs where disruption of a shared TFBS could confound interpretation of phenotypes. Accordingly, characterization of the mice reported here will require genetic complementation studies where either the TSPAN2 protein or the *Tspan2os* LncRNA are reconstituted to better interpret phenotypes. Based on natural genetic variation and in vivo mutagenesis screening in different mouse strains [42], we suspect there will be more examples of single-base edits in a TFBS resulting in attenuated target gene expression in mice.

The near loss in expression of *Tspan2* in smooth muscle tissues with a single-base pair edit of the CArG box was unexpected given the heterogeneity of SRF-binding CArG boxes across the genome [37]. This suggests that some substitutions across the CArG box are intolerant for SRF binding. Interestingly, despite the abundant expression of SRF in brain [43] and heart [44], the CArG box mutants generated here had little effect on expression of *Tspan2* in these tissues. This would imply that separable TFBS recognized by distinct transcription factors drive expression of *Tspan2* in heart and brain. Future studies could determine whether subtle editing of targeted TFBS around the *Tspan2* locus confer selective loss of expression in brain or heart.

Nearly all of our genome is noncoding, comprising tens of thousands of noncoding RNAs and millions of TFBS [3, 45]. Most SNVs associated with disease reside in noncoding sequence but causality of such regulatory SNVs in disease is notably lacking, especially in the complex milieu of an animal model [3]. For example, 146/164 (89%) coronary artery disease risk alleles harbor noncoding SNVs but, with the possible exception of the noncoding SNV near *SORT1* [46], there is a lack of insight into the functional consequence of such sequence variants in vivo. While no known SNV exists in the *Tspan2/Tspan2os* CArG box, the large number of such TFBS [35] would suggest the presence of potentially important CArG-SNVs that could be easily modeled with PE2 editing in the mouse. An important goal therefore will be to map all CArG-SNVs in the human genome and filter those of possible clinical relevance for further study.

A limitation of genome editing in animal model systems and in future clinical trials to correct disease-causing mutations is unintended editing at the desired editing site or at distal off-targets. Whole genome sequencing and screening experiments in animal models have demonstrated low-level off-targeting events with wild type Cas9 [47–50] and, more recently, prime editing [28] though it must be stressed that these studies were necessarily limited to very few guide RNAs. A broader analysis in mouse and rat showed measurable off-targeting in 23% of Cas9-sgRNA experiments, with most off-targeting nearly eradicated by high-fidelity Cas9 nucleases [51]. Two previous reports in mice have shown that the PE3 platform, which utilizes an sgRNA to create a second nick at the on-target site [24], elicits on-target indels and substitutions [28, 29]. Here, no on-target indels or substitutions above the limits of detection were found in 12 PE2 founder mice whereas unwanted on-target indels were detected in the majority of 11 HDR founders. The latter results are congruent with a pre-submission report in mouse embryos showing low on-target indels with the PE2 platform [29]. Although PE2 exhibited high-fidelity on-target editing at the *Tspan2* CArG box, the frequencies of correct editing were lower than that seen in the HDR arm of the study. This suggests a higher level of mosaicism that could reduce the efficacy of germline transmission. On the other hand, HDR founders showed varying degrees of on-target indels, which also contribute to mosaicism and challenges in germline transmission of the desired edit. Clearly, more studies in mice are needed to establish general rules regarding on-target editing efficiencies, extent of mosaicism, and efficacy in germ line transmission using different prime editing platforms.

An unbiased, genome-wide analysis using the recently developed CHANGE-seq protocol [39] identified nearly twofold greater number of candidate off-target sites with wild type Cas9 and sgRNA than wild type Cas9 and the pegRNA. The reason for the variance in candidate off-targets is unclear but may be due to technical reasons related to the different structures of the sgRNA and pegRNA. Interrogation of 244 candidate off-target sites revealed off-targeting events in 5/11 (45%) HDR founders. In contrast, we did not detect evidence of off-target mutations in PE2-edited founder mice, though definitive proof for the absence of off-targeting events will require whole genome sequencing studies. While the majority of off-targets predicted by CHANGE-seq (and CRISPOR) harbor a CArG box, bulk RNA-seq of aortic tissue failed to reveal reduced expression of any associated transcript. Moreover, the bulk RNA-seq data would suggest that the *Tspan2* CArG box acts locally to control the *Tspan2*/*Tspan2os* gene pair and not at a distal target gene. It will be of major interest to ascertain whether these observations extend to other CArG-dependent mRNA-LncRNA gene pairs. Importantly, the editing efficiencies and off-targeting reported here are based on a single protospacer. Additional studies analyzing more target sites with a broader spectrum of sequence edits are needed before general rules are established regarding the efficiency and fidelity of the PE2 system in mice.

## Conclusion

We have compared prime editing with HDR-mediated editing in mice and show the PE2 system is effective in the installation of a single nucleotide substitution within a TFBS without on-target indels or detectable off-targeting events. This single-base replacement confers a near complete loss in expression of the *Tspan2*/*Tspan2os* gene pair in smooth muscle-rich tissues, allowing for future characterization of phenotypes under baseline and stress-induced conditions. The tissue-restricted loss in *Tspan2* expression with subtle edits to a regulatory element suggests a new paradigm for generating cell-restricted knockout mice without the labor-intensive breeding associated with the *Cre*/*lox*P system. The PE2 platform comprises only two components, yielding precision-guided editing and minimal unwanted mutations. These desirable attributes, as well as the development of computational tools for optimal pegRNA design [52], should stimulate additional comparative studies to further assess the efficiency of prime editing in mice. Finally, it will be of great interest to assess the potential utility of the prime editing platform in somatic editing of both prenatal and postnatal animal models.

## Methods

### HDR-mediated genome editing of *Tspan2* CArG box in mice

The mouse experiments in this study were approved by local institutional animal care and use committees at Cornell University (#2000-0122) and Medical College of Georgia at Augusta University (#2019-0999 and #2019-1000). Fertilized oocytes derived from male B6(Cg)-Tyr^2J^/J (Jackson Laboratory, stock #000058) and superovulated female FVB/NJ (Jackson Laboratory, stock #001800) mice were microinjected with 50 ng/μl of wild type Cas9 mRNA (TriLink Biotechnologies, San Diego, CA), 50 ng/μl of sgRNA (Synthego Corp., Menlo Park, CA), and 25 ng/μl of ULTRAmer standard desalting ssODN (Integrated DNA Technologies, Coralville, IA) harboring a CCT>GTC substitution in the *Tspan2* CArG box (Fig. 1a). All CRISPR components were dissolved in nuclease-free water (Ambion #9932), diluted in injection buffer (100 mM NaCl; 10 mM Tris-HCl, pH, 7.5; and 0.1 mM EDTA), and injected into the pronucleus and cytoplasm of fertilized oocytes using a Nikon Eclipse TE200 microscope equipped with Eppendorf FemptoJet 4x, Eppendorf TransferMan NK manipulator, and Eppendorf CellTram Air vacuum (Enfield, CT). Injected embryos were cultured in KSOM medium at 37 °C overnight and viable two-cell staged embryos were transferred to the oviducts of pseudopregnant female mice of strain B6D2F1/J (Jackson Laboratory, stock #1000006) and allowed to develop to full term. Founder mice were weaned 21 days post-parturition and genomic DNA from tail snips isolated with Gentra Puregene Tissue Kit (Qiagen Sciences #158667; Germantown, MD) according to the manufacturer’s instructions. Allele-specific ssODN primers (Integrated DNA Technologies, Coralville, IA) were used to PCR genotype each founder pup for the presence of *Tspan2* CArG box editing. ssODNs for HDR-mediated repair and PCR genotyping are listed (Additional file 8: Table S7). Selected HDR founder mice were bred to strain C57BL/6J mice (Jackson Laboratory, #000664) to pass the CCT>GTC substituted CArG box allele through the germline for heterozygous intercrossing and gene expression analysis. In addition, 11 HDR founders were analyzed for on-target and off-target editing as described below.

### Prime editing of *Tspan2* CArG box in mice

The same strains of mice used in HDR-mediated editing were used for prime editing. The Cas9 nickase-reverse transcriptase plasmid (pCMV-PE2, Addgene #132775, Watertown, MA) was linearized with *PmeI* (New England Biolabs #R0560S, Ipswich, MA) for 3 h at 37 °C, excised from an agarose gel, purified with a Monarch® DNA Gel Extraction kit (New England Biolabs #T1020S, Ipswich, MA), and incubated with RNAsecure™ RNase Inactivation Reagent (Thermo Fisher Scientific #AM7005, West Columbia, SC) for 15 min at 65 °C. Linearized and purified pCMV-PE2 was then in vitro transcribed using mMESSAGE mMACHINE & Ultra kit (Thermo Fisher Scientific #AM1345, West Columbia, SC) for 3 h at 37 °C in the presence of RNasin Ribonuclease Inhibitor (Promega #N2111, Madison, WI), and PE2 mRNA was purified with MEGAclear™ Transcription Clean-Up kit (Thermo Fisher Scientific, #AM1908, West Columbia, SC) according to the manufacturer’s instructions. Pronuclear/cytoplasmic injections were carried out with 25 ng/μl each of the PE2 mRNA dissolved in RNAse-free water and the synthetic pegRNA dissolved in nuclease-free water and diluted in the same injection buffer used for HDR editing. Genomic DNA from tail snips of founder mice was isolated with Gentra Puregene Tissue Kit (Qiagen Sciences #158667; Germantown, MD) according to the manufacturer’s instructions and PCR genotyped with primers flanking the CArG box followed by restriction digestion of the PCR amplicon with *PflMI*. The latter restriction site (CCA [N]_5_TGG) is generated with installment of the C>G transversion. ssODNs for PE2-mediated repair and PCR genotyping are listed (Additional file 8: Table S7). Two PE2 founder mice were bred to strain C57BL/6J mice (Jackson Laboratory, #000664) to pass the C>G transversion allele through the germline for heterozygous intercrossing and gene expression analysis. In addition, 12 PE2 founders were analyzed for on-target and off-target editing as described below and two littermate controls exhibiting no editing (founders #32 and #38) were included for on-target editing efficiency only.

### Synthesis of pegRNA

The prime editing guide RNA (pegRNA) was synthesized using Synthego’s CRISPRevolution platform with solid-phase phosphoramidite chemistry. Based on the original prime editing report [24], we selected a reverse transcriptase (RT) template of 10 nucleotides in length, inclusive of the C>G transversion, and a primer binding site (PBS) of 16 nucleotides in length. Three 2′-O-methyluridinylates were attached at the 3′ end of the PBS and stabilized with phosphorothioate backbones. The first three bases of the protospacer were modified as 2′-O-methyl derivatives and stabilized as phosphorothioates. The pegRNA was purified using reversed-phase high-performance liquid chromatography (Buffer A, 0.1 M TEAA; Buffer B, 50% 0.1 M TEAA/50% acetonitrile, 15%–95% B gradient in 15 min), and their identities were confirmed using an Agilent 1290 Infinity II liquid chromatography system coupled with Agilent 6530B Quadrupole time-of-flight mass spectrometry (Agilent Technologies, Santa Clara, CA) in a negative ion polarity mode.

### Sanger sequencing

Initial PCR genotyping informed us of founder mice carrying either the three base-pair substitution (HDR) or the single- base substitution (PE2) in the *Tspan2* CArG box. PCR amplicons from several of these founders were prepared for cloning into the pCR4-TOPO TA vector (Thermo Fisher Scientific #450071) and plated on LB agar plates for ampicillin-resistant colony isolation, purification, and PCR validation with original primers to ensure the presence of a clone. Several independent clones from each founder were then prepared for Sanger sequencing (GENEWIZ®, Research Triangle Park, NC). Representative electropherograms were cropped in Adobe Photoshop for presentation.

### Quantitative RT-PCR analysis

Indicated tissues from adult mice were rapidly excised, cleaned of adhering tissue in ice-cold phosphate buffered saline, and plunged in liquid nitrogen. Tissues were homogenized with a Minilys homogenizer (Bertin Technologies, Rockville, MD) using a Precellys Lysing Kit (VWR Scientific, Radnor, PA). Total RNA was extracted from thoroughly homogenized tissues via miRNeasy Mini Kit (#217004, Qiagen) according to the manufacturer’s directions. The concentration of RNA was measured by a Nanodrop 2000 spectrophotometer (Thermo Fisher Scientific), and 200–500 ng of total RNA was programmed for cDNA synthesis using an iScript™ cDNA Synthesis Kit (#1708890 Bio-Rad; Hercules, CA). Universal SYBR Green Supermix (Bio-Rad)-based qRT-PCR was carried out in a CFX386 Touch™Real-Time PCR Detection System (Bio-Rad). *Tspan2* mRNA and *Tspan2os* RNA expression were calculated by the 2^−ΔΔCt^ method using *Hprt* as an internal housekeeping control. Primer sequences used in this study are listed (Additional file 8: Table S7). Expression data were derived from tissues of independent mice (sample sizes indicated in figure legends) of each genotype and each data point in the scatter plots represents the mean of technical replicates (*n* = 3) for each mouse.

### Immuno-RNA fluorescence in situ hybridization assay

Heart, brain, and aorta from *Tspan2*^*sg/sg*^ and *Tspan2*^*peg/peg*^ CArG mutants (and littermate controls) were fixed in 10% neutral buffered formalin, paraffin embedded, and cut at 5 μm. Sections were processed for combined immunofluorescence of LMOD1 protein (Proteintech, #15117-1-AP; 1:200 dilution), a specific marker for smooth muscle [34] and RNA in situ hybridization of *Tspan2* mRNA (RNAscope, ACD) according to the manufacturer’s instructions. Alexa fluor 488 secondary antibody was used to detect LMOD1 protein (Thermo Fisher). Signals were obtained with a LSM 900 confocal laser scanning microscope (Zeiss) using the Zeiss Blue software system for image acquisition and processing.

### Targeted sequencing analysis for on-target editing efficiency

Genomic DNA was isolated from the spleen of indicated HDR and PE2 founder mice by DNeasy Blood & Tissue Kit (QIAgen, #69504; Germantown, MD). Primers, with adapters for barcoding, were used to amplify 288 base pairs around the CArG box (Additional file 8: Table S7). 0.5 μL of PCR product was used as a template in a barcoding PCR reaction, consisting of 2 min at 98 °C, followed by 10 cycles of denaturation for 10 s at 98 °C, annealing for 20 s at 61 °C, extension for 30 s at 72 °C, and a final 2 min extension at 72 °C. Barcoded products were pooled and gel purified from a 1% agarose gel using a QIAgen kit (#28115) to remove primers before quantitation with a Qubit dsDNA HS Kit (Thermo Fisher Scientific #Q32851). Samples were loaded onto an Illumina MiSeq instrument with a 300 cycle v2 kit for sequencing. Greater than 30,000 reads were collected for each sample. Sequencing reads were demultiplexed using the MiSeq Reporter (Illumina) and fastq files were analyzed using Crispresso2 Batch Analysis [53]. An analysis window of 10 was used to identify indels. Analysis of some nuclease-treated mice yielded substantial fractions of non-aligning reads (1–79% of total reads). Visual inspection of these sequences in Geneious DNA analysis software (Biomatters Inc., San Diego, CA) indicated that they harbored larger (> 10 nt) deletions, so non-aligning reads were added to the quantified indels. Non-aligning reads were less than 1% of total reads for all prime-editor-treated mice.

### CHANGE-seq analysis for off-target events

Genomic DNA from B6(Cg)-Tyr^2J^/J (Jackson Laboratory, stock #000058) and FVB/NJ (Jackson Laboratory, stock #001800) mouse liver was purified using Gentra Puregene Kit (Qiagen) according to the manufacturer’s instructions and combined for CHANGE-seq as previously described [39]. Briefly, genomic DNA was tagmented with a custom Tn5-transposome to an average length of 400 bp, followed by gap repair with Kapa HiFi HotStart Uracil+ DNA Polymerase (KAPA Biosystems) and Taq DNA ligase (#M0208, New England Biolabs). Gap-repaired tagmented DNA was treated with USER enzyme (#M5508, New England Biolabs) and T4 polynucleotide kinase (#M0201, New England Biolabs). Intramolecular circularization of the DNA was performed with T4 DNA ligase and residual linear DNA was degraded by a cocktail of exonucleases containing Plasmid-Safe ATP-dependent DNase (Lucigen #E3101K), Lambda exonuclease (#M0262, New England Biolabs), and Exonuclease I (#M0293, New England Biolabs). In vitro cleavage reactions were performed with 125 ng of exonuclease-treated circularized DNA, 90 nM of SpCas9 protein (#M0386, New England Biolabs), NEB buffer 3.1, and 270 nM of sgRNA or pegRNA, in a 50 μL volume (please note: there is no purified Prime Editor protein at this time). Cleaved products were A-tailed, ligated with a hairpin adaptor (New England Biolabs), treated with USER enzyme (New England Biolabs) and amplified by PCR with barcoded universal primers NEBNext Multiplex Oligos for Illumina (#E7335, New England Biolabs), using Kapa HiFi Polymerase (KAPA Biosystems). Libraries were quantified by qPCR (KAPA Biosystems) and sequenced with 151 bp paired-end reads on an Illumina MiniSeq instrument. CHANGE-seq data analyses were performed using open-source CHANGE-seq analysis software (https://github.com/tsailabSJ/changeseq).

### Targeted sequencing by rhAmpSeq and indel analysis

On- and off-target sites for sgRNA and pegRNA targets were amplified from founder mouse spleen genomic DNA using two pools of customized rhAMPSeq libraries (Integrated DNA Technologies, Coralville, IA) (primers available upon request). Sequencing libraries were generated according to the manufacturer’s instructions and sequenced with 151 bp paired-end reads on an Illumina NextSeq instrument. Indel analyses were conducted using custom Python code and open-source bioinformatic tools. First, paired-end high-throughput sequencing reads were processed to remove adapter sequences with trimmomatic (version 0.36) [54], merged into a single read with FLASH (version 1.2.11) [55] and mapped to mouse genome reference mm10 using BWA-MEM (version 0.7.12) [56]. Reads that mapped to on-target or off-target sites were realigned to the intended amplicon region using a striped Smith–Waterman algorithm as implemented in the Python library scikit-bio; indels were counted and reported with total read counts.

### Bulk RNA-seq

RNA-seq and data analysis were performed in the Genome Research Center at the University of Rochester School of Medicine and Dentistry. Total RNA from individual aortae cleaned of periadventitial tissue was extracted and quantitated as described above and RNA quality assessed with the Agilent Bioanalyzer (Agilent, Santa Clara, CA). TruSeq-Stranded mRNA Sample Preparation Kit (Illumina, San Diego, CA) was used for next-generation sequencing library construction per the manufacturer’s protocols. Briefly, mRNA was purified with oligo-dT magnetic beads and fragmented for first-strand cDNA synthesis with random-hexamer priming followed by second-strand cDNA synthesis using dUTP incorporation. End repair and 3′ adenylation was performed on the double-stranded cDNA and Illumina adaptors were ligated, purified by electrophoresis, and PCR-amplified with primers to the adaptor sequences to generate amplicons of ~ 200–500 base pairs. Amplified libraries were hybridized to the Illumina flow cell and single-end reads of 75 nucleotides were generated using Illumina’s NextSeq550 sequencer (San Diego, CA). Raw reads were demultiplexed using bcl2fastq version 2.19.1. Quality filtering and adapter removal were performed using FastP (version 0.20.0) and cleaned reads were then mapped to *Mus musculus* (GRCm38 + Gencode-M22 Annotation) using STAR_2.7.0f. Gene level read quantification was derived using the subread-1.6.4 package (featureCounts) with a GTF annotation file (Gencode M22). Differential expression analysis was performed using DESeq2–1.22.1 with a *p* value threshold of 0.05 within R version 3.5.1 (https://www.R-project.org/). PCA plot, heatmap, and Gene Ontology analysis are available upon request. RNA-seq data have been deposited in the GEO database under accession number GSE158388 [38]. Scatter plots for log_10_ + 1 transformed reads of > 1 were generated in Excel for wild type control *Tspan2* CArG versus HDR-edited or PE2-edited *Tspan2* CArG box.

### Analysis of off-targets for CArG box and gene expression change by RNA-seq

Predicted off-targets from CRISPOR [33] and CHANGE-seq were interrogated for the presence of consensus CArG boxes (CCW_6_GG) or CArG-like boxes (consensus CArG box with 1 nucleotide substitution) and evidence of SRF-binding using data from ENCODE on the UCSC Genome Browser [57]. All predicted off-target sequences were then analyzed for the nearest transcription unit and these genes were cross-referenced to the RNA-seq data for changes in RNA expression.

### Statistics and data availability

All statistical analyses were conducted in GraphPad 8.0. We tested group values for normality with the Kolmogorov-Smirnov and Shapiro-Wilk tests. Differences in means (± standard deviation) were computed either with unpaired *t*-test for two comparisons or one-way ANOVA followed by Tukey’s post hoc testing for more than two comparisons. Statistical significance was assumed with a probability value of *p* < 0.05. All data generated or analyzed during this study are included in this published article and its supplementary information files or deposited in a public database (GSE 158388).

## Supplementary Information

**Additional file 1.** Supplementary figures.

**Additional file 2.** Supplementary Table S1. CRISPR targets.

**Additional file 3.** Supplementary Table S2. Dseq of bulk RNA-seq.

**Additional file 4.** Supplementary Table S3. Pool 1 off-targets.

**Additional file 5.** Supplementary Table S4. Pool 2 off-targets.

**Additional file 6.** Supplementary Table S5. sgRNA off-targets and CArG boxes.

**Additional file 7.** Supplementary Table S6. pegRNA off-targets and CArG boxes.

**Additional file 8.** Supplementary Table S7. List of primers.

**Additional file 9.** Review history.

## Data Availability

The datasets used and/or analyzed during the current study are available from the corresponding authors on reasonable request, including original bulk RNA-seq data under GSE158388 [38].
