# Supplementary material for: Prime editing in mice reveals the essentiality of a single base in driving tissue-specific gene expression
Source: Genome Biol. 2021 Mar 16;22:83. doi: 10.1186/s13059-021-02304-3 (PMC7962346; doi:10.1186/s13059-021-02304-3)
Supplement: Supplementary file 1 — Additional file 1. Supplementary figures. [file 13059_2021_2304_MOESM1_ESM.pdf]

**a**

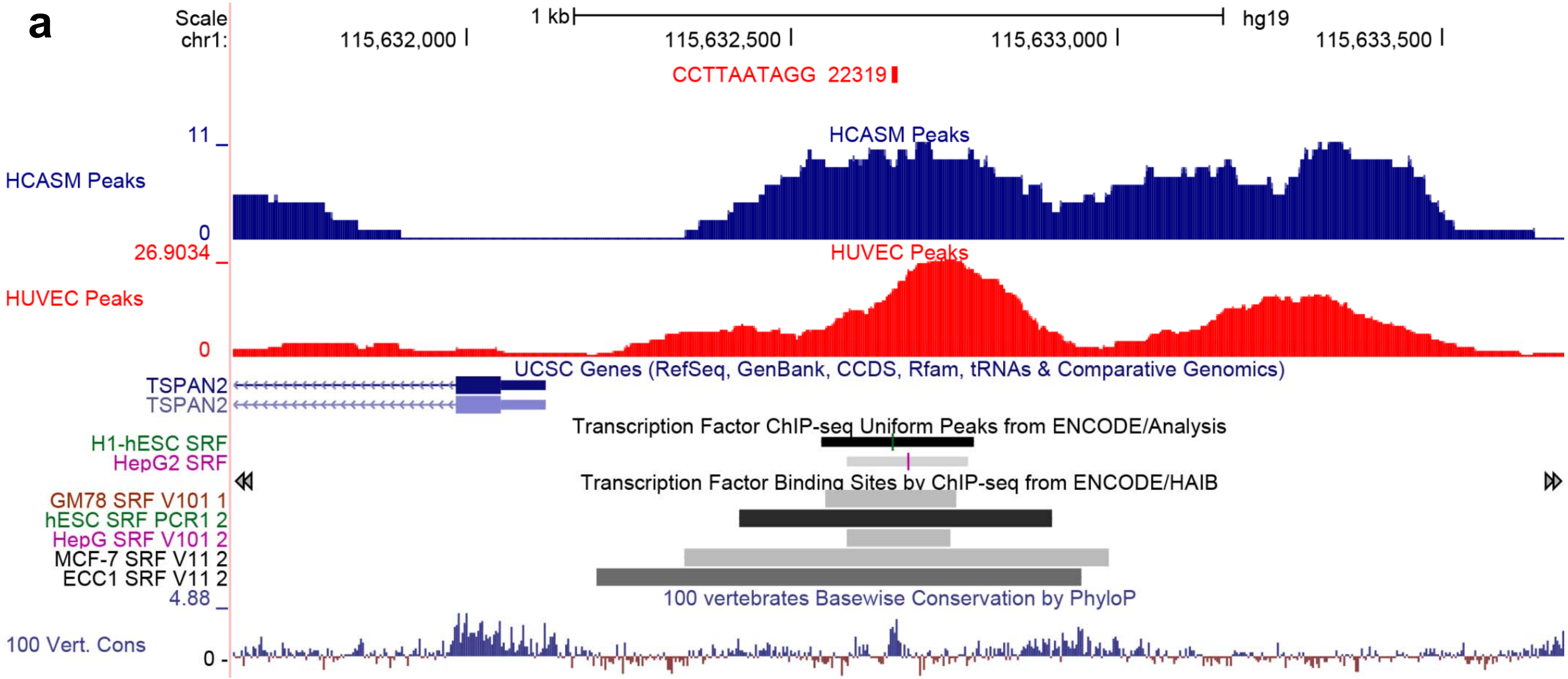


**b**

| | |
|---|---|
| Human | CTCTTACCTTAATAGGCAAGAA |
| Chimp | CTCTTACCTTAATAGGCAAGAA |
| Rhesus | CTCTTACCTTAATAGGCAAGAA |
| Mouse | CTGTTACCTTAATAGGCAAGAA |
| Rat | TTGTTACCTTAATAGGCAAGAA |
| Rabbit | CTCTTACCTTAATAGGCAAGAA |
| Pig | CTCTTACCTTAATAGGCAAGAA |
| Dolphin | CTCTTACCTTAATAGGCAAGAA |
| Dog | CTCTTACCTTAATAGGGCAGAA |
| Elephant | CTGTTACCTTAATAGGCAAGAA |
| Opossum | TCCTTACCTTAATAGGCATGAA |

**Supplementary Figure 1. Human *TSPAN2* locus and CArG sequence.**
(**a**) Screenshot of UCSC Genome Browser showing CArG sequence in 5' promoter region (red at top) and ChIP-seq data for SRF-binding in human coronary artery SMC (blue) and human umbilical vein endothelial cells (red). Also shown are SRF ChIP-seq data from ENCODE ( dark bars). Note the *TSPAN2* locus is transcribed from the Crick strand in this view. There is no annotated lncRNA associated with the *TSPAN2* locus in human (see Fig. 4a for mouse lncRNA,*Tspan2os*).
(**b**) Conservation of CArG sequence (red) is shown to left with PAM sequences highlighted yellow; the PAM sequence utilized in this study is to left (see Fig. 1a, 3a). Sequence divergence flanking CArG box is indicated with green nucleotides.

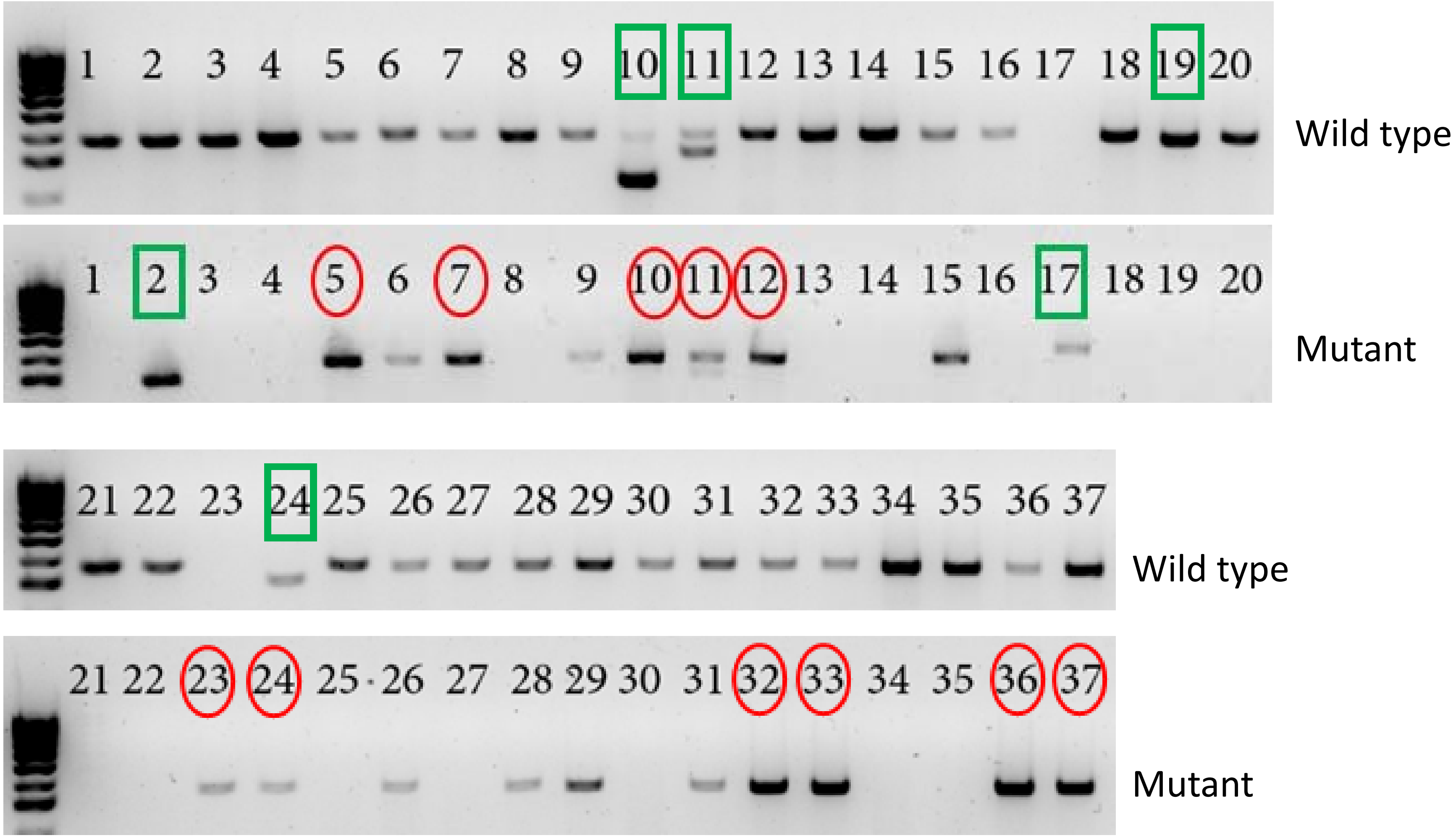


. . .CCTATTAAGGTAACAGTCCTCTAG. . . CArG wild type
. . .**<u>GTC</u>**ATTAAGGTAACAGTCCTCTAG. . . CArG Mutant

**Supplementary Figure 2. Genotyping of original founder mice derived from HDR editing of *Tspan2* CArG box**. Primers specific to the wild type sequence or the 3 base pair substitution (CCT > GTC) were used in separate PCR reactions to generate the indicated bands above. Those founders boxed in green indicate the presence of obvious indels. Those founders circled in red represent the mutants analyzed for further study. Sequences below represent wild type and mutant (bold underlined) CArG boxes. Founders 33-37 are also shown in Fig. 1b of the main text.

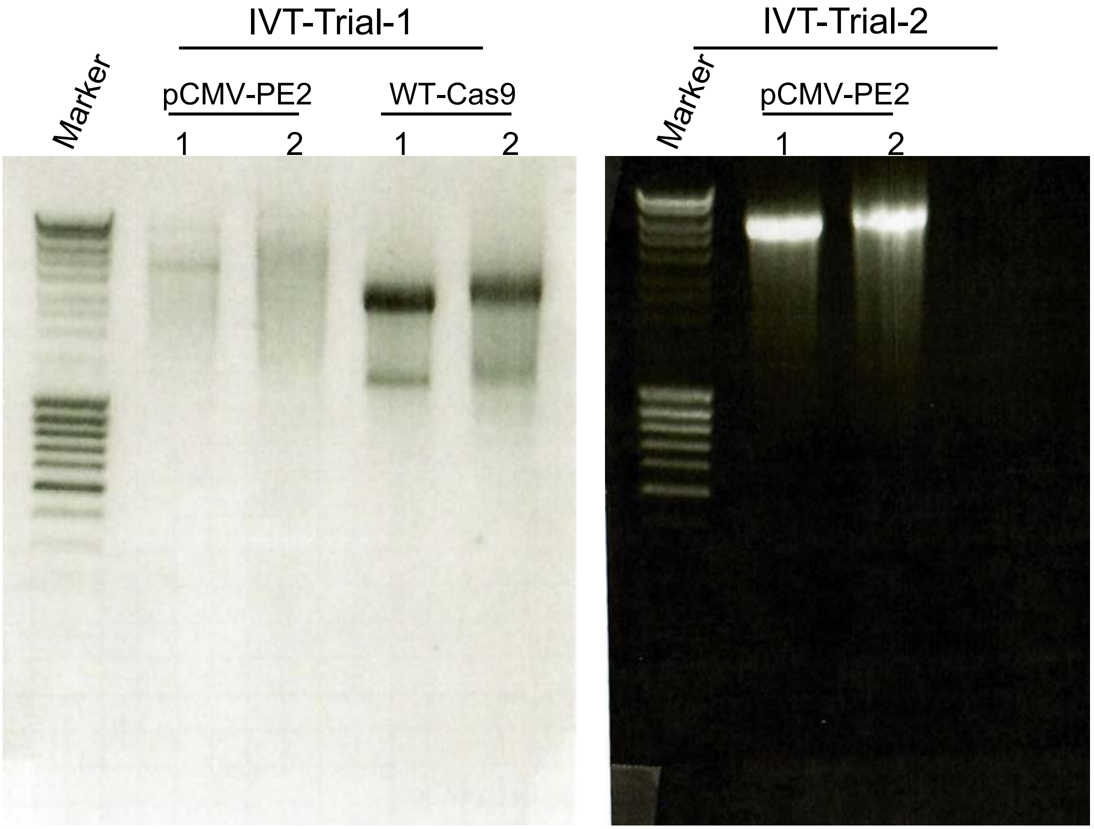


**Supplementary Figure 3. Optimized in vitro transcription of PE2 plasmid**. The pCMV-PE2 plasmid was initially In vitro transcribed (IVT) alongside wild type Cas9 with standard conditions per manufacturer (Trial 1). Poor quality PE2 mRNA necessitated a prolonged IVT reaction (3 hrs) and the addition of RNAse inhibitors (Trial 2). The latter conditions resulted in higher quality mRNA for microinjections. Lane 1 samples were non-polyadenylated and lane 2 samples were polyadenylated yielding a slightly slower mobility band.

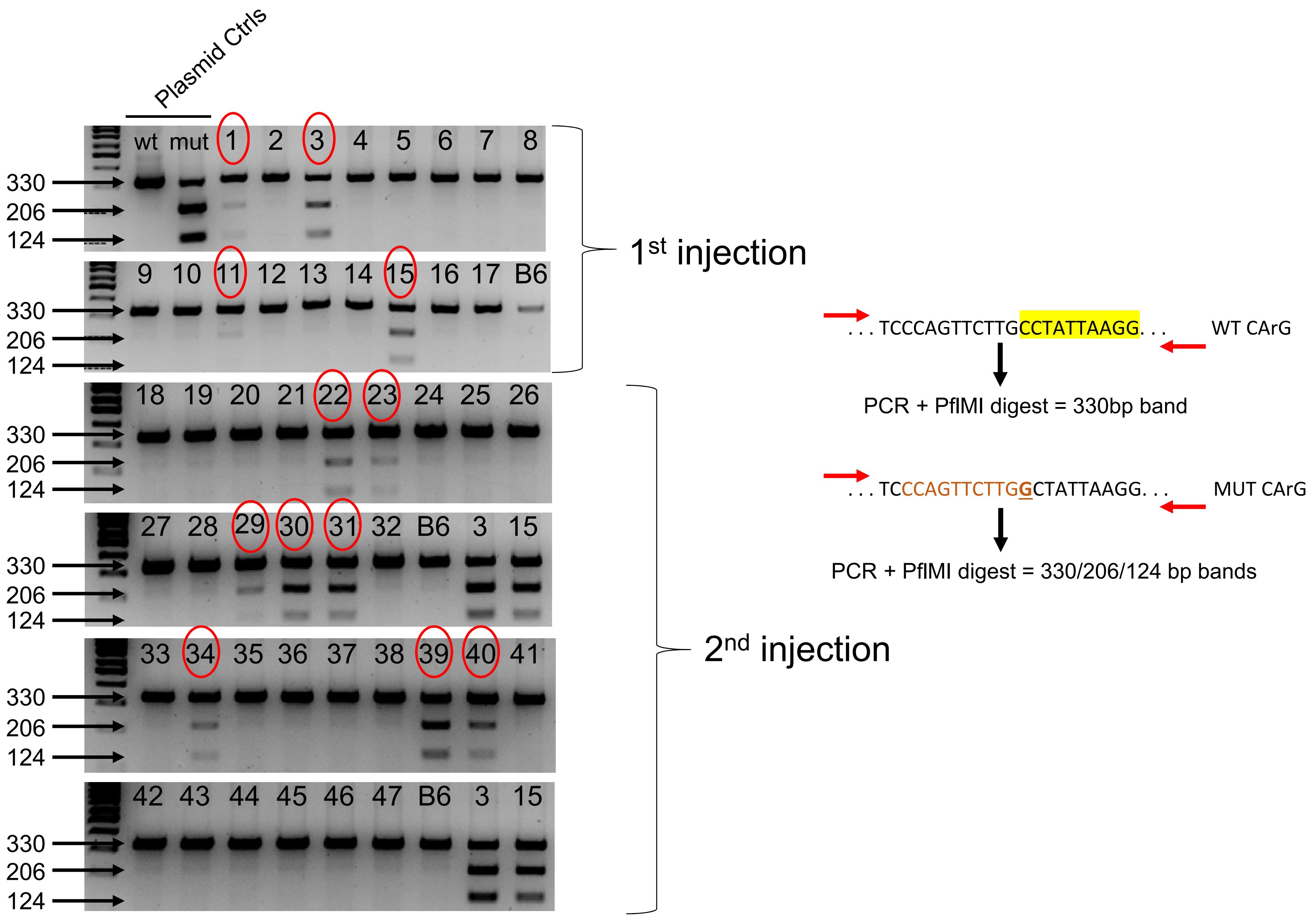


**Supplementary Figure 4. Genotyping of original founder mice from PE2 editing of *Tspan2* CArG box**. A 330 base pair PCR amplimer was subjected to PflMI restriction digestion (right). The recognition sequence for this enzyme (CCA[N]$_5$TGG) is generated with a C>G transversion (bold underlined MUT CArG). Founders circled in red denote those mice used for further analysis. A B6 wild type mouse was used as a negative control and founders 3 and 15 from injection 1 were used as positive controls in founder geno-typing of the second microinjection. The CArG box at top right is shaded yellow. Founders 28-32 are also shown in Fig. 3b.

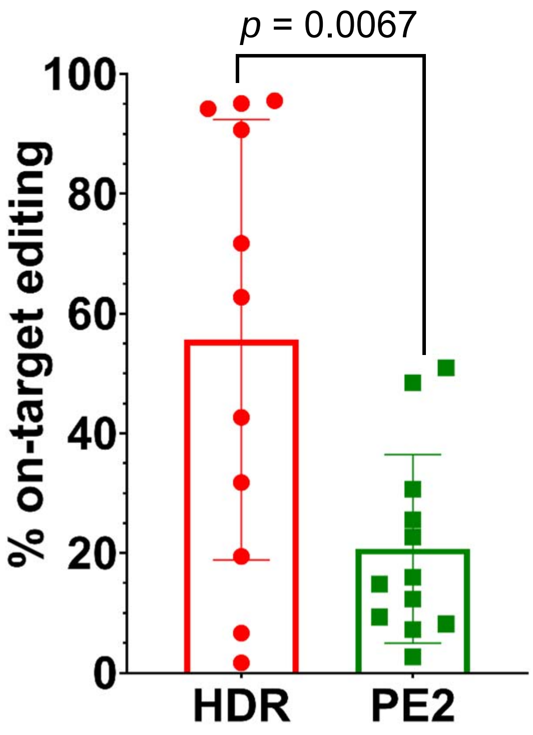


**Supplementary Figure 5. On target editing efficiencies in HDR and PE2 founder mice**. Scatter plots of the percent desirable editing in HDR (sgRNA) versus PE2 (pegRNA) founder mice. Each data point represents the percentage of reads in an individual founder mouse exhibiting the correctly edited CArG box.

## Predicted guide sequences for PAMs

Ranked by default from highest to lowest specificity score (Hsu et al., Nat Biot 2013). Click on a column title to rank by a score.
If you use this website, please cite our paper in NAR 2018. Too much information? Look at the CRISPOR manual.

Download as Excel tables: Guides / Guides, all scores / Off-targets / Saturating mutagenesis assistant

| Position/ Strand | Guide Sequence + *PAM* + Restriction Enzymes ☐ Only G- ☐ Only GG- ☐ Only A- | MIT Specificity Score | CFD Spec. score | Predicted Efficiency (Show all scores) | | Outcome | | Off-targets for 0-1-2-3-4 mismatches + next to PAM | Genome Browser links to matches sorted by CFD off-target score ☐ exons only ☐ chr3 only |
|---|---|---|---|---|---|---|---|---|---|
| | | | | Doench '16 | Mor.-Mateos | Out-of-Frame | Lindel | | |
| 50 / fw | TCCTCTAGGAACGCAGCGGC *AGG*<br>Enzymes: *MspA1I, TauI, Fsp4HI*<br>**Cloning / PCR primers** | 88 | 93 | 46 | 45 | 74 | 65 | 0 - 0 - 0 - 3 - 73<br>0 - 0 - 0 - 0 - 0<br>76 off-targets | 4:intron:Frmd4b<br>4:intergenic:SNORA3-U6<br>4:exon:Ivl<br>show all... |
| 46 / fw | ACAGTCCTCTAGGAACGCAG *CGG*<br>Enzymes: *MspA1I, TauI, ApeKI, Fsp4HI, BstV1I*<br>**Cloning / PCR primers** | 85 | 85 | 71 | 66 | 66 | 83 | 0 - 0 - 1 - 8 - 155<br>0 - 0 - 0 - 0 - 0<br>164 off-targets | 4:intron:Wdr75<br>3:intron:Ubp1<br>4:intergenic:Gm8526-Edil3<br>show all... |
| 14 / rev | TAGAGGACTGTTACCTTAAT *AGG*<br>Enzymes: *Tru1I*<br>**Cloning / PCR primers** | 80 | 93 | 18 | 77 | 74 | 77 | 0 - 1 - 0 - 12 - 68<br>0 - 0 - 0 - 1 - 1<br>81 off-targets | 4:exon:2810021B07Rik<br>4:intron:Fam38b<br>4:intergenic:Phactr2-Fuca2<br>show all... |
| 31 / rev | TCCTGCCGCTGCGTTCCTAG *AGG*<br>Enzymes: *HpyCH4III, MaeI*<br>**Cloning / PCR primers** | 78 | 84 | 60 | 30 | 81 | 86 | 0 - 0 - 1 - 21 - 179<br>0 - 0 - 0 - 0 - 1<br>201 off-targets | 3:intergenic:U6-AC167724.1<br>4:intron:Igsf21<br>4:intergenic:Il12b-Gm12157<br>show all... |

**Supplementary Figure 6. Specificity score and off-target profile of protospacer used in HDR and PE editing of *Tspan2* CArG box.** The yellow highlighted cell indicates the protospacer sequence used in this study. Shown are the MIT and CFD specificity scores; higher scores (0-100) indicate a more specific protospacer for targeted sequence. Note the low number (81) predicted off targets for this protospacer. A full listing of these off-target sequences and a hyperlink to the UCSC Genome Browser is presented in Table S1.
