# Supplementary material for: Prime editing in mice reveals the essentiality of a single base in driving tissue-specific gene expression
Source: Genome Biol. 2021 Mar 16;22:83. doi: 10.1186/s13059-021-02304-3 (PMC7962346; doi:10.1186/s13059-021-02304-3)
Supplement: Supplementary file 9 — Additional file 9. Review history. [file 13059_2021_2304_MOESM9_ESM.docx]

The manuscript had previously been reviewed at another journal. We obtained a reviewer to assess the revisions, and the authors’ responses.

**Reviewer 1**

The manuscript by Gao et al. compared the editing characteristic of HDR and prime editing (PE) in mice, and indicated that the PE2 induces efficient single base changes with high fidelity. Meanwhile, the study also showed that the prime editing (PE2) exhibited high specificity and did not induce off-target genome-wide using CHANGE-seq. The authors have addressed all the comments from the two previous reviewers. To further improve the manuscript, I have some comments for this study.

1. This study only used one target site to compare the HDR and PE editing efficiencies in mice. It must be difficult to make a conclusion because PE exhibits low editing efficiencies at many target sites. I recommend to delete the comparison.

2. The PE2 platform has already been established in adult mice (Liu et al., BioRxiv 2020). The manuscript did not represent the first work in demonstrating prime editing in adult mouse using PE2 system. The authors should cite this article, and revise relevant statements in the manuscript.
